# Ethyl methanesulfonate mutagenesis in fungi: genetic mechanisms, applications, and implications for agricultural biotechnology

**DOI:** 10.1007/s00203-026-04858-x

**Published:** 2026-04-20

**Authors:** Darin Edward Holman, Ameerah Imaan Abrahams, Kacey Hattingh, Crishé Saulse, Miché Hess, Daegan Stegmann, Muizz Roomanay, Gerhard Basson, Ashwill Klein, Marshall Keyster

**Affiliations:** 1 Environmental Biotechnology Laboratory, Department of Biotechnology, University of the Western Cape, Bellville, 7535 South Africa; 2 Plant Omics Laboratory, Department of Biotechnology, University of the Western Cape, Bellville, 7535 South Africa

**Keywords:** Biocontrol agents, Ethyl Methanesulfonate mutagenesis, Fungal genetics, Mutant libraries, Sustainable agriculture

## Abstract

Ethyl methanesulfonate (EMS) is a widely used chemical mutagen that induces high-frequency point mutations and has been extensively applied in genetic studies across diverse biological systems. While EMS-based mutagenesis frameworks are well established in plant research, their application in fungal systems remains comparatively fragmented and methodologically inconsistent. This review synthesizes current knowledge on the mechanisms, efficiency, and mutation profiles of EMS in fungi, with emphasis on forward genetics, functional genomics, strain improvement, and pathogenicity studies relevant to agricultural biotechnology. We critically evaluate methodological limitations, mutation validation challenges, and gaps in standardised screening and sequencing pipelines relative to plant EMS platforms. Furthermore, we contextualize reported laboratory-scale trait enhancements, such as enzyme production and stress tolerance, within their prospective agricultural relevance. Overall, this review highlights the need for standardized fungal EMS workflows, integrated genomic validation, and ecologically relevant phenotyping to advance fungal biotechnology and agricultural applications.

## Introduction

The application of chemical mutagens in genetic research has unfolded over several decades, demonstrating significant potential in enhancing crop traits and elucidating basic biological processes. As far back as the mid-twentieth century, researchers began exploring various mutagens, including both physical agents such as gamma rays and chemical agents like ethyl methanesulfonate (EMS). EMS, a powerful alkylating agent, has emerged as one of the most widely utilized chemical mutagens due to its ability to induce site-specific point mutations, particularly in guanine bases, leading to high-frequency mutagenesis and effective genetic variation (Shirasawa et al. 2016; Li et al. 2017).

Historically, EMS was first recognized for its potent mutagenic properties in the 1950s. Since then, it has been extensively employed in numerous plant systems, including tomatoes, soybeans, cotton, and tetraploid durum wheat, to develop mutant populations used in both functional genomics and crop improvement programs (Lian et al. 2020; Chen et al. 2023; Di Sotto et al. 2012; Capilla-Perez et al. 2018). Its appeal lies in its cost-effectiveness, standardized protocols, and relatively straightforward application, making it accessible and adaptable across a range of experimental designs (Unan et al. 2022).

Despite its widespread use in plant systems, the application of EMS-induced mutagenesis in fungi remains comparatively underexplored. Fungi, however, hold immense ecological and agricultural relevance, contributing to nutrient cycling, soil health, plant growth promotion, and biological control of pests and diseases. They also play an expanding role in industrial biotechnology. The underrepresentation of fungal systems in EMS research may stem from limited genomic tools and a lack of standardized methodologies for many non-model fungal species (Gu et al. 2020). Nevertheless, growing interest in fungal biotechnology underscores the need for deeper investigation into the mutagenic potential of EMS in these organisms, particularly for enhancing beneficial traits, studying pathogenicity, and discovering novel secondary metabolites (Groppi et al. 2024; Shiwa et al. 2012).

In this context, EMS mutagenesis shows strong potential to stand as a valuable and widely used technique in advancing fungal genetic research, offering a powerful platform for both forward and reverse genetics. EMS acts primarily as an alkylating agent, inducing random point mutations, particularly G/C to A/T transitions, that can lead to phenotypic diversity useful for dissecting gene function. Next-generation sequencing technologies have streamlined the detection and mapping of EMS-induced mutations, increasing the efficiency and precision of mutant identification (Zhang et al. 2014). Such integration facilitates high-throughput screens and functional genomics pipelines, particularly in the construction of mutant libraries and mapping populations (Papdi et al. 2010).

In forward genetic frameworks, EMS has enabled the identification of novel mutants with enhanced agronomic traits, including stress resistance, improved yield, and adaptive metabolic responses (Lunde et al. 2024). In fungi, these mutations can be harnessed to modify enzyme profiles, improve host–pathogen interactions, or modulate virulence, ultimately supporting sustainable agriculture and biotechnology (Wang et al. 2022; Calderini et al. 2011). However, challenges remain, such as the potential for off-target effects, non-uniform mutation distribution, and the need for bioinformatics pipelines to filter causal variants (Guo et al. 2024b). Recent efforts incorporating machine learning and genomic profiling have begun to address these limitations.

Despite increasing interest in fungal biotechnology, the application of EMS mutagenesis in fungi remains fragmented, with limited methodological standardisation and very few studies linking mutation, phenotype, and agricultural outcomes. Current literature is dominated by isolated strain-improvement reports, with little synthesis across taxa or trait categories, and limited integration with agricultural performance frameworks. Building robust EMS mutant libraries in fungi holds the potential to unlock new genetic insights, facilitate strain improvements, and address critical agricultural issues such as disease resistance and environmental resilience (Saito et al. 2011; Susrama et al. 2022).

Multiple physical and chemical mutagenesis approaches are available for generating genetic variation in microorganisms, including ultraviolet (UV) irradiation, gamma irradiation, ion beam irradiation, and chemical mutagens such as methyl methanesulfonate (MMS) and N-methyl-N-nitrosourea (MNU), which are highlighted by Holman et al. (2025) in Table 1 for bacterial strains. Physical mutagens often induce broad DNA damage, including large deletions and chromosomal rearrangements, but are frequently associated with higher lethality and genomic instability. In contrast, EMS predominantly induces point mutations, particularly G:C to A:T transitions, enabling dense allelic variation with greater reproducibility, and cost-effectiveness (Udagawa et al. 2021). While targeted genome editing tools such as CRISPR-Cas systems offer precise mutagenesis, their application in many filamentous and non-model fungi remains limited by low transformation efficiencies, heterokaryosis, and species-specific genetic constraints (Hassane et al. 2025). EMS represents a practical and scalable approach for generating genetic diversity in genetically under-resourced fungal systems.

**Table 1:** Overview of EMS mutagenesis conditions and their effects on various fungal species

Organism	Mutagen	Concentration (%)	Exposure time (min)	Survival/lethality (%)	Mutation detection method	Screening throughput	Stability testing duration	Genetic validation of phenotype	References
Soil fungal isolate	EMS	5	60	NR	Enzyme activity assays (lipase activity)	NR	NR	No	Sharma et al. 2019
Ashbya gossypii	EMS	5	90	NR	Extracellular enzyme activity assays (amylase, β-glucosidase)	Moderate (multiple mutants screened)	NR	No	Ribeiro et al. 2013
Thermomyces dupontii	EMS (with physical mutagenesis context)	0.1	30	NR	Enzyme activity assays + 18S rDNA identification	~ 40 mutants screened (moderate)	NR	Partial (strain ID only, not mutation-level)	Nisar et al. 2020
Beauveria bassiana	EMS	0.5	60	NR	Phenotypic assays (growth, spore germination, pathogenicity tests)	Low–moderate (few isolates screened)	5–15 days recovery after heat exposure	No	Wongwanich et al. 2017
Alternaria citri	EMS	0.5	60	NR	Enzymatic assays + kinetic analysis (Km, Vmax)	Moderate (multiple EMS concentrations and mutant screening)	NR	No	Ahmed et al. 2025
Penicillium janthinellum	EMS + UV	0.17	1440	NR	Plate clearance assays + cellulase activity assays (CMCase, FPase)	Moderate (successive selection on cellulose media)	NR	No	Adsul et al., 2007
Aspergillus oryzae	EMS	0.14	720	NR	Bioactivity screening (antibacterial assays against pathogens)	High (> 3000 EMS-treated cultures screened)	NR	No	Leonard et al. 2013
Penicillium roquefortii	EMS + UV	10	30–60	NR	Metabolite profiling + lipase activity assays	Moderate (mutants grouped by metabolite and enzyme profiles)	NR	No	El-Bondkly & Keera 2007
Volvariella volvacea	EMS (combined with UV, γ-ray, electron beam)	1	60	~ 70–80% lethality optimised	Phenotypic selection + genome shuffling screening	Moderate (16 mutants used in shuffling pool)	Multiple rounds with stability assessment of selected strains	No (phenotypic stability only)	Zhu et al. 2016
Penicillium oxalicum (OXPoxGA15A)	EMS + ARTP (multiple rounds)	1.2	480 (8 h)	NR	RT-qPCR + genomic re-sequencing (SNP detection) + enzyme assays	Very high (3532 mutant colonies screened)	NR (repeated cultivation implied)	Yes (gene expression and SNP validation)	Gu et al. 2020

The table summarizes effective EMS concentrations and exposure times that led to notable phenotypic changes, including enhanced enzyme activity and pathogenicity. These findings highlight the potential of EMS mutagenesis for strain improvement and functional genomic applications in fungi

NR = Not reported in the original study. Screening throughput categories were derived from the number of mutants screened where explicitly stated. Genetic validation refers to molecular confirmation (e.g., sequencing, SNP analysis, or gene expression), not phenotypic assays alone

This review therefore consolidates current knowledge on EMS mutagenesis in fungi, critically evaluates methodological advances, and identifies key gaps limiting its agricultural application, with emphasis on mechanistic insights, optimisation strategies, and phenotypic screening frameworks. Although EMS-derived traits such as enhanced enzyme production and stress tolerance may have agroecological relevance, most evidence remains laboratory-based, and field-scale resilience outcomes are still underexplored. Accordingly, the review focuses on experimentally supported applications and prospective agricultural implications rather than definitive field-validated resilience claims. While the focus is on fungal EMS mutagenesis, selected examples from plants, bacteria, and other microbial systems are included for methodological comparison where fungal-specific data remain limited. These comparative studies are not presented as directly equivalent to fungal pipelines, but rather as conceptual frameworks that inform experimental design, mutation density optimisation, and genomic validation strategies. Fungal systems exhibit distinct biological features, including heterokaryosis, variable ploidy, complex life cycles, and often limited transformation efficiency, that differentiate them from plant mutant-library infrastructures and model bacterial systems (Meyer et al. 2016). Consequently, the transferability of approaches such as TILLING, high-throughput mutant libraries, and large-scale sequencing workflows must be interpreted cautiously and adapted to fungal genetic and physiological constraints.

## Ethyl Methanesulfonate (EMS): a versatile mutagen in genetic fungal studies

### Mechanisms of EMS-induced mutagenesis

Ethyl methanesulfonate (C_3_H_8_O_3_S) is regarded as one of the most prevalent alkylating mutagens due to its high efficiency and mutagenicity (Singh et al. 2021; Türkoğlu et al. 2023). This chemical mutagen has diverse effects on DNA, as it induces a high density of random single base point mutations across microbial genomes, including fungi (Espina et al. 2018).

Enzyme-mediated biological reactions often hinge on the reactivity of specific nucleophilic sites within cellular molecules. EMS exerts its mutagenic activity primarily due to its electrophilic nature, which allows it to transfer ethyl groups to various nucleophilic sites in proteins and nucleic acids, potentially leading to mutagenesis. Nucleophilic sites within biomolecules are commonly found in the side chains of amino acids such as cysteine, lysine, and histidine, or within the nucleobases of DNA and RNA (Netto et al. 2007).

DNA possesses nucleophilic sites primarily within its bases, notably at the N7 position of guanine and the N3 position of adenine. These sites can exhibit significant nucleophilic activity, enabling mutagens like EMS to form adducts that disrupt normal base pairing during DNA replication (Wyatt and Pittman 2006). Such modifications could lead to mispairing and potentially generate mutations, illustrating how nucleophilic modifications can promote genomic instability.

Transitional states in biochemical reactions are crucial, particularly concerning DNA repair and replication (Stivers and Jiang 2003). EMS alkylates nucleophilic sites on DNA, potentially inducing aberrant transitions that disrupt normal replication. The presence of adducts can obstruct the function of DNA polymerases, resulting in replication errors that manifest as mutations and repair accuracy (Chatterjee and Walker 2017).

**Fig. 1:**
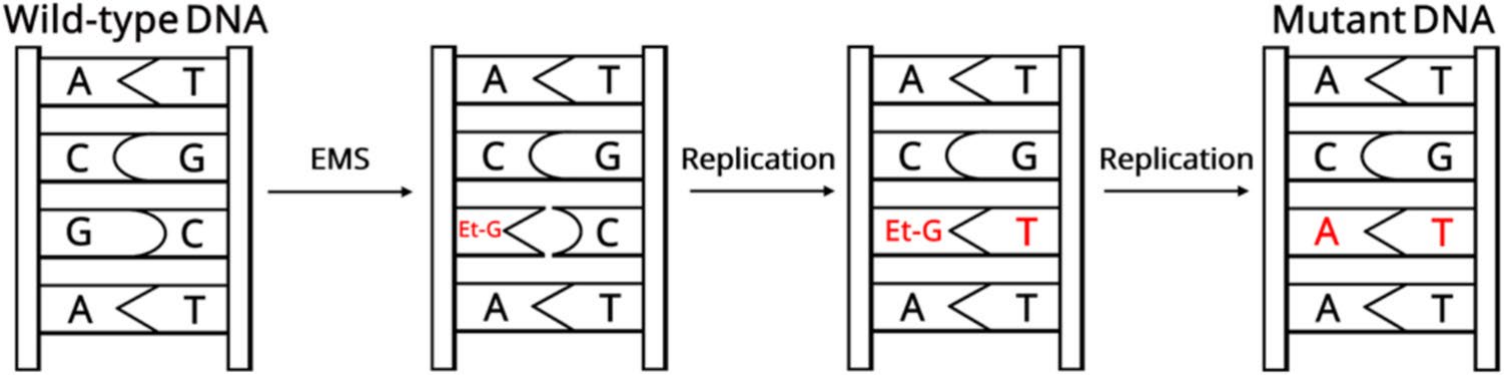
EMS-induced transition mutation mechanism. EMS alkylates guanine (G), forming O6-ethylguanine (Et-G), which mispair with thymine (T) instead of cytosine (C) during DNA replication. Subsequent replication leads to a permanent transition mutation from G/C to A/T in the DNA sequence

This results in a spectrum of G:C-to-A:T transitions (Fig. 1), leading to either non-synonymous or synonymous mutations due to EMS-induced DNA lesions, base modifications, or nucleotide substitutions. These transitions primarily occur due to guanine alkylation at the O6 or N7 positions (Snyman 2022). Specifically, as illustrated in Fig. 1, the formation of O6-ethylguanine disrupts normal hydrogen bonding with cytosine, making it compatible with thymine instead. Consequently, during DNA replication, thymine is incorporated, and subsequent DNA repair mechanisms detect and excise O6-ethylguanine, ultimately leading to an A:T base pair formation. The characteristic G:C to A:T transition can manifest at the translation level and lead to altered protein function, stability, or expression, ultimately generating phenotypic diversity in subsequent generations (Zafar et al. 2024). Consequently, due to its base-specific reactivity and ability to generate a high density of mutations, EMS-induced mutagenesis is a powerful tool for functional genomic studies and trait improvement.

### Mutagenic potency and reactivity of EMS as an alkylating agent

Within experimental mutagenesis frameworks, both physical and chemical mutagens have been employed to induce genetic variation in microbial and fungal systems, each differing in mutation spectrum, cytotoxicity, and genomic consequences (Hoyos-Manchado et al. 2020; Shrivastav et al. 2010). Physical mutagens such as ultraviolet (UV), gamma irradiation, and ion beam irradiation generate heterogeneous DNA lesions, including strand breaks, oxidative damage, and chromosomal rearrangements, which can produce high-impact mutations but are frequently associated with elevated lethality, genomic instability, and complex mutation profiles that complicate downstream genetic analysis (Szakmary et al. 1996; Hoyos-Manchado et al. 2020). Ion beam mutagenesis has gained attention as a high-energy irradiation technique capable of inducing diverse mutation types, including large deletions and genomic rearrangements, and has been successfully applied in microbial and plant mutagenesis studies (Guo et al. 2024a). In contrast, chemical mutagens, particularly alkylating agents such as methyl methanesulfonate (MMS), N-methyl-N-nitrosourea (MNU), and EMS, induce DNA base alkylation, resulting in more defined mutation spectra determined by their reaction mechanisms and adduct formation patterns (Shrivastav et al. 2010; Wyatt and Pittman 2006).

Among alkylating mutagens, EMS is widely regarded as a highly effective S_N2-type ethylating agent that primarily targets nucleophilic sites on DNA, especially the N7 position of guanine and, to a lesser extent, the O^6^ position, generating adducts such as 7-ethylguanine and O^6^-ethylguanine (Shrivastav et al. 2010; Wyatt and Pittman 2006). The mutagenic activity of EMS is largely attributable to O^6^-ethylguanine, which mispairs with thymine during DNA replication and leads predominantly to G:C to A:T transition mutations, a mutation spectrum that is well documented across microbial and fungal model systems (Taira et al. 2013; Hoyos-Manchado et al. 2020). Compared to methylating agents such as MMS, which predominantly produce N-methyl adducts associated with cytotoxicity and replication blockage, EMS induces a higher proportion of heritable point mutations while maintaining comparatively moderate cytotoxicity (Shrivastav et al. 2010; Wyatt and Pittman 2006).

Relative to highly potent S_N1 alkylating agents such as MNU, which generate elevated levels of O^6^-alkylguanine and exhibit substantial cytotoxic and clastogenic effects, EMS typically produces a more balanced profile of mutagenicity and cellular survival, allowing efficient recovery of viable mutant populations (Shrivastav et al. 2010; Wyatt and Pittman 2006). Furthermore, the predominance of point mutations and the relatively low frequency of large-scale chromosomal rearrangements distinguish EMS from physical mutagens such as gamma irradiation, which frequently induce double-strand breaks and recombinogenic events that can compromise genome integrity and obscure genotype–phenotype relationships (Szakmary et al. 1996; Hoyos-Manchado et al. 2020).

From a fungal genetics perspective, these properties confer several practical advantages. EMS is cost-effective, reproducible, and readily standardised across experimental systems, enabling controlled mutagenesis and the generation of dense allelic variation suitable for forward genetic screening and mutant library construction (Hoyos-Manchado et al. 2020; Meyer et al. 2016). Many filamentous and non-model fungi exhibit low transformation efficiencies, heterokaryotic growth, and species-specific genetic constraints that limit the application of targeted genome-editing approaches. In such systems, EMS mutagenesis provides a scalable and experimentally tractable strategy for inducing genome-wide point mutations while preserving overall genome structure, thereby facilitating downstream mutation mapping, functional characterisation, and strain improvement (Meyer et al. 2016; Hoyos-Manchado et al. 2020). The predictable mutation spectrum, moderate cytotoxic profile, and ease of experimental optimisation underpin the continued prominence of EMS as a preferred mutagen in fungal genetic studies and biotechnological strain development.

### EMS efficiency and mutation profiles

EMS has gained prominence as a preferred mutagen in biotechnology due to its efficiency in inducing genetic diversity. In bacteria, EMS-induced mutants of *Bacillus licheniformis* and *Brevibacillus borstelensis* exhibited enhanced antimicrobial activity against the pathogens *Staphylococcus aureus* and *Salmonella typhi*, respectively (Kortam et al. 2023). Similarly, in microalgae, EMS treatment of *Chlorella pyrenoidosa* generated mutant strains with significantly improved biomass and lipid productivity, key traits for biofuel production (Vani et al. 2023). These studies confirm the robustness of EMS as a mutagenic tool for developing strains with improved industrial traits, ranging from antibiotic production in bacteria to improved biofuel precursors in algae.

EMS-induced mutagenesis is also a standard tool in mycology, applied across yeasts and filamentous fungi. For instance, studies on the model yeast *Saccharomyces cerevisiae* have used EMS to enhance tolerance to inhibitory compounds present in industrial fermentations. Kadeba and Wilgers (2020) generated EMS-mutagenized yeast with improved tolerance to high ethanol concentrations, while Wawro (2021) developed a mutant strain with increased acetic acid resistance and a 60% higher ethanol yield (120 g/L) than the parental strain. Such strains are vital for optimising industrial bioethanol production. In filamentous fungi, EMS mutagenesis has likewise proven effective in enhancing enzyme production. For example, mutagenesis of *Alternaria citri* (Ahmed et al. 2025) and *Penicillium funiculosum* NCIM 1228 (Chavan et al. 2024) yielded hyper-cellulase producing mutants. The enhanced cellulolytic potential of these strains enables more efficient hydrolysis of lignocellulosic biomass waste for applications in enzyme production, biofuel synthesis, and waste degradation. While applications in yeast and filamentous fungi are well-documented, the use of EMS in dimorphic, pathogenic, and higher fungi (e.g., basidiomycetes and mushrooms) remains a significant knowledge gap, despite its demonstrated potential. For instance, Wongwanich et al. (2017) used EMS to induce thermotolerance in the entomopathogen *Beauveria bassiana*. One mutant isolate, BCNT002MT, demonstrated superior growth, sporulation, and pathogenicity against the brown planthopper (*Nilaparvata lugens*) at elevated temperatures (33 °C), highlighting its promise as a more resilient biological control agent. In the king oyster mushroom, *Pleurotus eryngii*, EMS mutagenesis generated strains with improved high-temperature tolerance and enhanced xylanase and cellulase activities (Romruen et al. 2021). These traits can lead to higher yields and reduced energy costs during spawn running, offering advantages for industrial cultivation. The limited research in these ecological and industrial important fungal groups represents a critical frontier for future EMS-based studies, which could elucidate mutational tolerance and repair mechanisms across the fungal kingdom.

While EMS mutagenesis is a powerful and universal tool for generating genetic diversity across multiple microbial systems, its random mutagenic nature can introduce unintended secondary mutations, necessitating extensive high-throughput screening to identify rare beneficial mutants. Furthermore, excessive DNA alkylation inflicted by EMS may overwhelm repair pathways, resulting in cytotoxicity and lethal mutations. These limitations highlight the need for meticulous species-specific optimisation of EMS parameters (e.g., concentration and exposure time), which will be discussed in the following section.

The studies in Table 1 show that EMS commonly enhances hydrolytic enzyme secretion, particularly within cellulase and xylanase classes, under relatively moderate EMS concentrations and short exposure times. Trait improvements are more consistent for metabolic outputs than for pathogenicity or ecological resilience, indicating that enzyme production is more directly selectable under laboratory conditions. Several studies also report parallel gains in stress tolerance, suggesting a potential link between metabolic enhancement and adaptive performance.

### Leveraging EMS in forward genetics: creating and identifying novel mutants

**Fig. 2:**
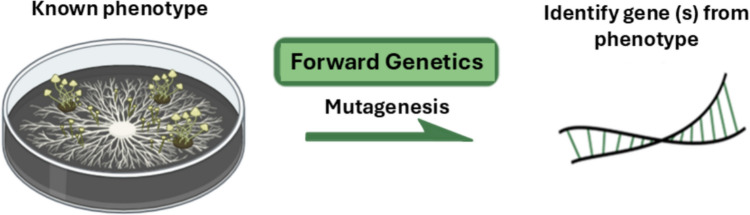
Overview of the forward genetics approach. This technique begins with a phenotype of interest and uses random mutagenesis, followed by phenotypic screening, to identify mutants with altered traits. Subsequent genetic analysis is then used to pinpoint the causal gene(s) underlying the observed phenotype. This phenotype-to-gene approach enables the discovery of genetic determinants without prior knowledge of gene function

Forward genetics links a novel phenotype to its underlying genetic determinant by screening mutagenised populations for altered traits Fig. 2. In fungi, EMS remains one of the most widely applied chemical mutagens due to its ability to induce dense, genome-wide G/C → A/T transitions at high frequency (Sega 1984; Greene et al. 2003). This enables rapid generation of mutant populations that can be screened for agriculturally relevant traits such as altered enzyme secretion, stress tolerance, metabolic output, or pathogenicity (Kangara et al., 2021; Afifi et al. 2014; Elakkiya & Muralikrishnan 2014).

In fungal biotechnology, EMS-based forward screens have successfully identified mutants with enhanced cellulase activity, thermotolerance, and modified virulence profiles (Elakkiya and Muralikrishnan 2014; Wongwanich et al. 2017; Abdullah et al. 2013). For example, in *Beauveria bassiana*, EMS mutagenesis facilitated discovery of thermotolerant and more virulent phenotypes relevant to biological pest control (Wongwanich et al. 2017). These studies demonstrate how phenotype-first screening enables functional inference even in genetically under-resourced fungi.

Forward EMS screens therefore provide a practical entry point for trait discovery and strain development, particularly in non-model species lacking robust genetic tools (Salazar-Cerezo et al. 2023).

#### Advantages of creating and identifying novel mutants in forward genetics

One of the most significant advantages of forward genetics techniques is their unbiased nature, which requires no prior knowledge concerning a specific biological pathway. Furthermore, mutagens can cause a wide range of mutations, reducing lethality of the organism (Khan et al. 2021). It also encompasses tools such as random mutagenesis and adaptive laboratory evolution, which enable the generation of large pools of mutant phenotypes without requiring prior knowledge of the organism’s genetics or metabolism. These approaches are often more cost-effective and less time-consuming than developing new molecular tools, which can be both labour-intensive and expensive (Trovão et al. 2022).

**Fig. 3:**
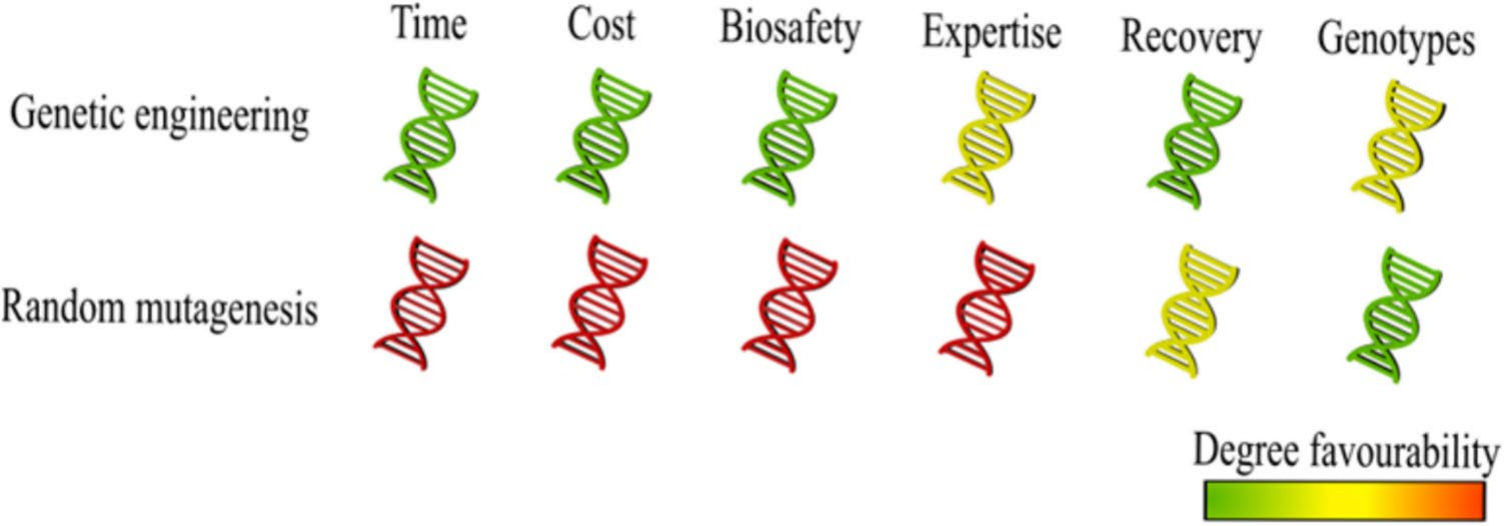
Strain improvement approaches by forward and reverse strategies. Comparison of several aspects of three methods of strain improvement: random mutagenesis, adaptive laboratory evolution and genetic engineering

Treatment with EMS is cost-effective, simple to implement, and triggers point mutations at rapid rates and with consistent results in most species that have various genetic backgrounds (Fig. 3) (Tsuda et al. 2015; Gady et al. 2009). EMS mutagenesis can produce a significant amount of mutants in a brief amount of time, which may promote studies of stress tolerance in plants (Chen et al. 2023), compared to physical mutagens, they are less expensive and simpler to utilize (Martín et al. 2009). These advantages also underpin the use of EMS as a complementary tool within reverse genetic studies.

#### Screening approaches

Forward genetic screening is an important gene discovery tool for matching a trait of interest to the causative gene. Forward genetic screens often expand a population's genetic variety by chemical mutagenesis, gamma irradiation, or random genome-wide incorporation of transgenes along with transposons. Mutagenesis is subsequently followed by selecting the mutant lines with the desired phenotype. Induced mutagenesis produces a considerably broader spectrum of mutations than spontaneous mutagenesis since many mutations are chosen against in natural populations (Javorka et al. 2019).

#### Identification and application

Efficient mutation identification is critical for both expediting molecular breeding and performing functional analyses on mutant genes. If the gene sections of a mutant's whole genome have already been sequenced, any mutations in the library may be found without a screening phase, significantly speeding up reverse genetics and its use in plant breeding. Mutations can be discovered more thoroughly by utilizing whole-genome sequencing (WGS) and whole-exome sequencing (WES) with target-capturing panels. Mutations were discovered in sorghum (*Sorghum bicolor*) by the WGS analysis of 256 EMS mutant lines, and in wheat (*Triticum aestivum*) by WES analysis of 2735 EMS mutant lines respectively. Every mutation in these mutant libraries has been catalogued (Udagawa et al. 2021).

EMS-induced mutagenesis is a standard approach in forward genetics, providing a reliable way for producing and detecting new mutants. Its applications encompass functional genomics, crop improvement, and model organism research, adding greatly to our understanding of gene function and the production of useful features (Simons et al. 2022; Martín et al. 2009).

### Best practices for EMS mutagenesis in fungal systems

The effective application of EMS mutagenesis in fungal systems requires standardised experimental design that accounts for organism-specific biology, mutation burden, and reproducibility constraints. A critical first step is empirical optimisation of EMS dosage through kill-curve analysis, in which a range of concentrations and exposure durations are tested to establish a dose–response relationship and target survival rates that balance mutation density with cellular viability (Chhaya and Gupte 2019; Hu et al. 2017; Kurowska et al. 2011). Optimal survival thresholds are context-dependent; stringent forward genetic screens may employ lower survival rates to recover rare mutants, whereas strain improvement studies often favour intermediate survival to preserve physiological fitness (Serrat et al. 2014; Shreya et al. 2019).

Fungal-specific biological features, including multinucleate hyphae, heterokaryosis, ploidy variation, and complex life cycles, can mask recessive mutations and complicate genotype–phenotype interpretation. Accordingly, mutagenesis of uninucleate cells such as spores or protoplasts, followed by single-spore isolation and purification of homokaryotic lines, is recommended to improve phenotypic consistency and genetic resolution (Hu et al. 2017; Kurowska et al. 2011; Serrat et al. 2014). Adequate mutant population sizes, often comprising hundreds to thousands of isolates, and biological replication across independent screens are essential to capture rare mutations and reduce stochastic variation (Chhaya and Gupte 2019; Hu et al. 2017).

To minimise confounding effects of passenger mutations, integration of genomic validation strategies such as whole-genome sequencing, backcrossing where sexual cycles permit, and complementation analysis is strongly encouraged (Hu et al. 2017; Ilarduya et al. 2001; Chhaya & Gupte 2019). In addition, systematic stability testing across multiple generations and culture conditions should be implemented to assess phenotypic robustness and reversion risks. Transparent reporting of key parameters—including EMS concentration, exposure time, quenching method, survival rate, target cell type, mutant population size, phenotype definition, and validation strategy—is essential for cross-study comparability and reproducibility in fungal EMS workflows (Chhaya and Gupte 2019; Słoczyńska et al. 2014).

### Leveraging EMS in reverse genetics: functional dissection of genes and pathways

Reverse genetics is fundamentally a genotype-driven approach that investigates gene function by examining phenotypic consequences of targeted genetic perturbation, typically through gene editing, gene replacement, or gene silencing strategies (Singh et al. 2021). Unlike these targeted approaches, EMS mutagenesis is a random mutagenesis strategy and does not constitute a reverse genetics method per se. However, EMS can complement reverse-genetic objectives in fungal systems by generating allelic series, partial loss-of-function variants, and conditional phenotypes, particularly in organisms where targeted genome editing remains inefficient or technically constrained (Snyman et al. 2021; Wang et al. 2024). This is especially relevant in filamentous and non-model fungi, which often exhibit low transformation efficiencies, heterokaryosis, and species-specific barriers to CRISPR-Cas systems and homologous recombination-based approaches (Zou et al. 2021; Yang et al. 2024). In such contexts, EMS-derived mutants can facilitate functional gene analysis when complete knockouts are lethal or genetically inaccessible, thereby supporting reverse-genetic investigations without replacing targeted methodologies (Chen et al. 2023; Snyman et al. 2021).

While reverse genetics traditionally relies on precise gene-editing tools, such as gene replacement or CRISPR-Cas systems, these approaches are not universally effective on fungi (Yang et al. 2024). Many filamentous fungi exhibit low transformation efficiencies, poor homologous recombination rates, and species-specific barriers that limit the application of targeted genome modification (Zou et al. 2021). Additionally, knockouts of essential genes often kill the organism, preventing functional analysis through complete gene disruption (Wirtz et al. 2024). Thus, highlighting the need for alternative approaches that can disrupt gene function without killing the organism, especially in fungi that are difficult to manipulate genetically.

Within this context, EMS mutagenesis, although classically associated with forward genetic screening, has become a valuable supporting tool for reverse genetic applications in fungi. EMS induces high-frequency, reproducible point mutations that generate a spectrum of allelic variants across the genome including partial-loss-of-function alleles (Snyman et al. 2021). Such alleles are particularly useful when complete knockouts are lethal or when CRISPR-mediated editing cannot be applied effectively (Wang et al. 2024). By enabling the recovery of viable mutants that retain partial gene function, EMS provides a means to probe essential genes, dosage- sensitive pathways, and regulatory elements that cannot be interrogated through null mutations (Chen et al. 2023). EMS does not replace traditional reverse-genetic technologies but rather extends them, enabling the functional dissection of genes and pathways in fungi that are difficult to genetically manipulate.

#### Advantages of creating and identifying novel mutants using reverse genetics

**Fig. 4:**
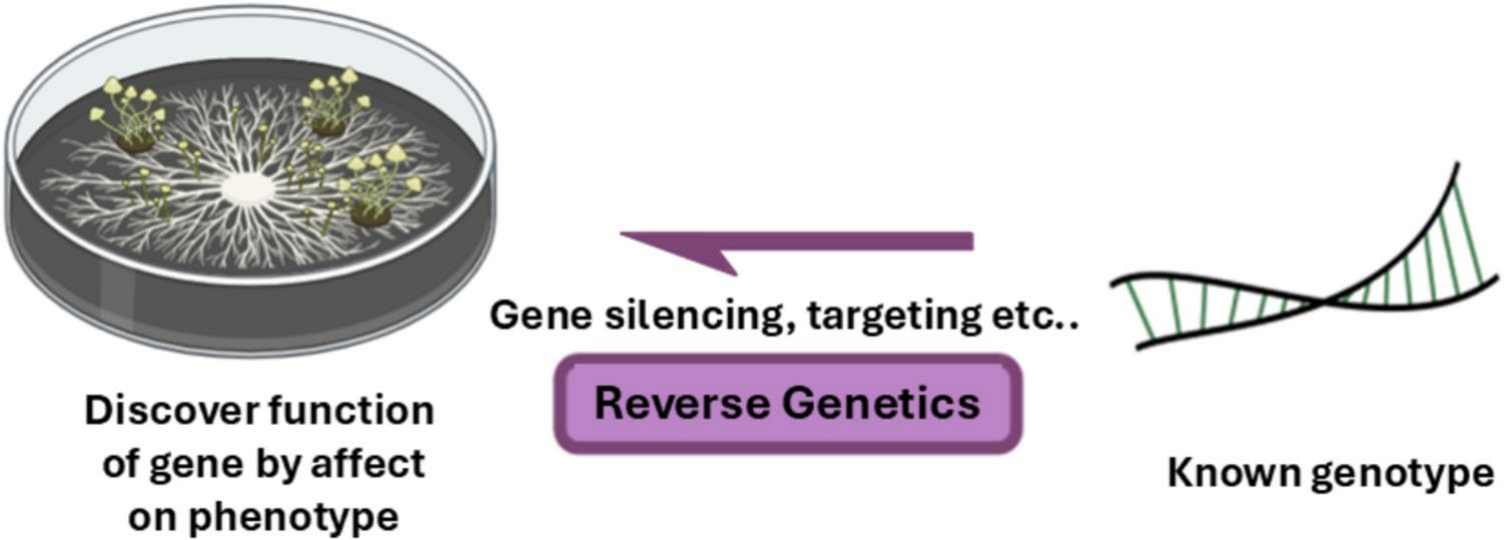
Overview of the reverse genetics approach. This approach begins with a known gene or genetic locus and employs targeted genetic manipulation, such as gene silencing, knockout, or directed mutagenesis, to assess its functional role. By observing the resulting phenotypic changes, researchers infer how disruption of the specific genotype affects organismal traits, thereby enabling gene-to-phenotype functional characterization

Reverse genetics enables targeted investigation of gene function by linking specific genotypes to defined phenotypic effects (Akilu, 2021; Singh, 2020). Although CRISPR and gene replacement remain core tools, low transformation efficiencies and essential-gene lethality often limit their application in fungi (Yang et al. 2024; Zou et al. 2021; Wirtz et al. 2024). In this context, EMS mutagenesis provides a practical complement by generating partial-loss-of-function alleles that preserve viability while still perturbing gene activity (Snyman et al. 2021). This strategy is particularly valuable when complete knockouts are lethal or when CRISPR-mediated editing cannot be applied efficiently (Wang et al. 2024; Chen et al. 2023), thereby extending the scope of functional gene analysis in filamentous fungi (Fig. 4).

#### Screening approaches

To identify mutations in EMS-mutagenized fungal populations, various screening strategies are employed. These include silencing strategies, screening populations for insertion, deletion, and point mutations, as well as gene targeting and ectopic expression using transgenic techniques (Ben-Amar et al. 2016). Recent advancements in reverse genetics have introduced RNA interference (RNAi) and Targeting Induced Local Lesions in Genomes (TILLING) as effective screening methods (Aklilu 2021). CRISPR-based gene editing also serves as a powerful tool for targeted mutagenesis and functional gene analysis (Shah et al. 2015).

TILLING is particularly advantageous due to its high-throughput nature and broad applicability across different organisms. This technique uses mismatch-specific enzymes to identify mutations in target genes through heteroduplex analysis, providing a cost-effective method for detecting single base changes (Aklilu 2021).

#### Identification and application

The successful application of EMS mutagenesis requires careful consideration of key parameters, such as the uniformity of the organism to be mutated and the optimization of EMS dosage (Chen et al. 2023). Next-generation sequencing (NGS) technologies have revolutionized the identification of EMS-induced mutations, offering high-throughput and accurate detection of genetic alterations (Thole and Strader 2015). The functional characterization of these mutations provides valuable insights into gene regulation, protein interactions, and metabolic pathways in fungi.

Reverse genetics has been widely applied in pathogenicity studies, where mutations affecting virulence factors in fungal pathogens are identified, contributing to the development of novel disease control strategies (Meng et al. 2023). Additionally, this approach has been employed in improving fungal strains for industrial applications, such as biofuel production (Adegboye et al. 2021) and biocontrol agents (St. Leger and Wang 2010), underscoring its versatility and importance in biotechnology research.

In conclusion, reverse genetics serves as a valuable and widely used technique of modern molecular biology, offering a precise and efficient approach to investigate gene function. By integrating EMS mutagenesis with advanced screening and molecular characterization techniques, researchers can unlock new insights into fungal biology, paving the way for innovative applications in sustainable agriculture and biotechnology.

### Identification of causal mutations in EMS-mutagenised fungi: sequencing and validation pipeline

The identification of causal mutations in EMS-mutagenised fungal strains is inherently challenging due to the high density of background (passenger) mutations introduced by alkylation-based mutagenesis. Consequently, a structured fungal-focused validation pipeline is required to distinguish driver mutations from non-causal variants. A recommended workflow begins with primary phenotypic screening of large mutagenised populations, followed by whole-genome sequencing (WGS) of selected mutants and comparison against the parental reference genome to catalogue induced single nucleotide polymorphisms (Hu et al. 2017; Kurowska et al. 2011). Where sexual cycles are available, backcrossing to the wild-type strain can be used to segregate passenger mutations and progressively enrich for the causal allele across generations (Chhaya and Gupte 2019).

In fungi lacking convenient sexual reproduction, alternative strategies include sequencing multiple independent mutants with similar phenotypes, functional annotation-based variant prioritisation, and complementation analysis to confirm genotype–phenotype relationships (Ilarduya et al. 2001; Hu et al. 2017). Additional validation approaches may include segregation analysis, allele replacement, or CRISPR-based functional confirmation in genetically tractable species. Importantly, fungal-specific factors such as heterokaryosis, epistatic interactions, and phenotypic instability can obscure causal inference and should be controlled through single-spore isolation, purification of homokaryotic lines, and repeated phenotypic verification across passages (Stonesifer and Baltz, 1985; Meyer et al. 2016).

The integration of WGS, genetic segregation, and functional validation provides a robust framework for causal variant identification in EMS-derived fungal mutants, particularly in non-model species with complex genomes, limited transformation efficiency, and fragmented genetic resources (Meyer et al. 2016; Hu et al. 2017). This pipeline reduces false attribution of phenotypes to passenger mutations and improves the reliability, reproducibility, and interpretability of forward genetic studies in fungal systems.

**Fig. 5:**
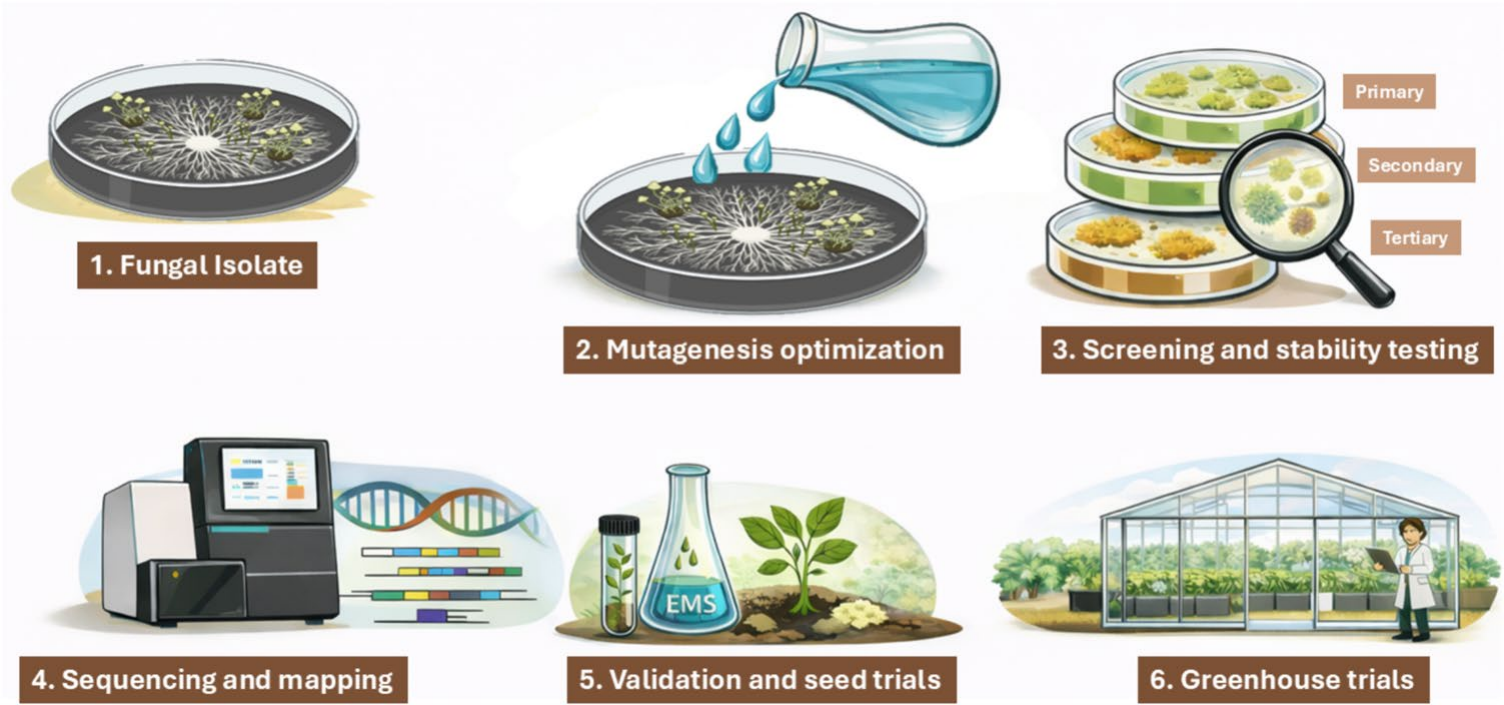
Schematic workflow of EMS mutagenesis in fungal systems for agricultural biotechnology. The figure illustrates a conceptual pipeline including fungal isolate selection, mutagenesis optimisation, tiered screening and stability testing, sequencing and mutation mapping, validation of improved traits, and greenhouse-level evaluation prior to agricultural application

A consolidated schematic workflow for fungal EMS mutagenesis in agricultural biotechnology, encompassing mutagenesis optimisation, multi-tier screening, sequencing and mapping, validation, and greenhouse-level testing, is presented in Fig. 5 to improve methodological clarity and cross-study comparability.

### Limitations and challenges of EMS-induced mutagenesis

Although EMS mutagenesis is a widely used technique for generating genetic diversity, it is not without its challenges. One of the primary concerns is the stability of induced mutations, as these alterations can revert to wild-type alleles over time, complicating the isolation of stable mutant strains. This highlights the need for robust selection methods to maintain phenotypic stability and ensure the persistence of beneficial traits. Several factors influence mutant stability, including genomic context, genetic background, and environmental conditions. For instance, certain genomic regions may be more prone to reversion events, while the genetic background of an organism can affect the manifestation and viability of induced mutations. Additionally, environmental factors may further impact the stability of mutant phenotypes.

To mitigate these challenges, selective strategies such as backcrossing, genetic complementation, and molecular screening are essential for preserving stable mutant phenotypes and minimizing reversion risks. Moreover, continuous monitoring of mutant populations is crucial for detecting early signs of reversion and implementing corrective measures. Since some mutations may result in lethality or reduced fitness, effective selection strategies must be in place to isolate viable mutants with desirable traits. The integration of advanced screening techniques, such as CRISPR/Cas9 genome editing, fluorescence-activated cell sorting (FACS), and next-generation sequencing, can enhance the efficiency of mutant identification and characterization. These high-throughput approaches not only improve the precision of selection but also facilitate the long-term stability of beneficial mutations (Garrido-Cardenas et al. 2018; Ren et al. 2019; Nguyen et al. 2024).

Thus, while EMS mutagenesis remains a valuable tool for genetic modification, addressing the challenges of mutation stability and reversion is essential for its effective application. By employing rigorous selection strategies and incorporating advanced screening technologies, researchers can enhance the reliability and utility of this technique in fungal mutagenesis and genetic studies.

## Applications of EMS fungal mutants towards sustainable agriculture

In the context of fungal agricultural biotechnology, resilience-related performance can be defined as the capacity of a fungal strain to maintain functional activity under variable and often suboptimal environmental conditions relevant to agricultural systems. Rather than representing a single trait, resilience encompasses a suite of measurable characteristics including stress tolerance (temperature, osmotic, UV, and pH), rhizosphere competence, persistence in soil environments, competitive fitness against native microbiota, and consistency of functional traits such as antagonism, enzyme production, or plant growth promotion across fluctuating environmental conditions (Meyer et al. 2016).

However, most EMS-derived fungal studies evaluate phenotypes under controlled in vitro conditions, which may not reliably predict performance in complex soil, rhizosphere, or field environments. To improve translational relevance, a tiered evaluation framework is warranted. Initial laboratory assays should include stress tolerance profiling, growth kinetics under abiotic stress, and assessment of phenotypic stability across successive passages. These should be complemented by greenhouse-level evaluations assessing rhizosphere colonisation, plant–fungus interaction outcomes, and functional consistency under variable moisture, temperature, and soil types, followed where feasible by soil microcosm or field-relevant assessments of persistence, ecological fitness, and sustained functional performance (Meyer et al. 2016). The adoption of multi-environment phenotyping frameworks would reduce overreliance on laboratory-only phenotypes and enable more robust evaluation of agriculturally relevant traits in EMS-derived fungal strains.

Despite increasing application of EMS mutagenesis in fungi, the quality and comparability of evidence across agricultural application domains remain heterogeneous. Many studies rely on phenotype-first screening under controlled laboratory conditions, often with limited mutant population sizes, replication, and long-term validation, which constrains statistical power and introduces bias toward easily screenable traits such as enzyme activity, stress tolerance, or morphology (Hoyos-Manchado et al. 2020; Meyer et al. 2016). Moreover, mutation burden is rarely quantified, and the integration of whole-genome sequencing, backcrossing, or complementation analyses to confirm causal genotype–phenotype relationships remains inconsistent, increasing the risk of confounding by background (passenger) mutations (Hoyos-Manchado et al. 2020; Wyatt and Pittman 2006; Kubo et al. 2022). Phenotypic stability is also infrequently assessed beyond short-term laboratory passages, leaving reversion risks and long-term robustness of EMS-induced traits insufficiently characterised (Hoyos-Manchado et al. 2020; Taira et al. 2013). Collectively, these limitations highlight the need for more standardised fungal EMS workflows integrating defined screening frameworks, genomic validation, stability testing, and ecologically relevant phenotyping to improve reproducibility and agricultural translatability.

From an applied perspective, EMS mutagenesis has demonstrated potential for enhancing fungal traits relevant to sustainable agriculture, including hydrolytic enzyme production, stress tolerance, and functional interactions with plants and soil microbiota. Enhanced secretion of hydrolytic enzymes may accelerate organic matter turnover and nutrient mineralisation, thereby supporting plant nutrient availability and reducing reliance on synthetic fertilisers. Similarly, mutants exhibiting increased thermotolerance, osmotic stress resistance, or oxidative stress resilience may maintain functional activity under climate-driven environmental fluctuations, which is critical for sustainable biocontrol and soil amendment strategies.

Importantly, EMS mutagenesis generates incremental allelic variation that can modulate, rather than abolish, fungal ecological interactions. Such subtle genetic variation is particularly relevant for improving biocontrol robustness, rhizosphere competence, and symbiotic performance, where ecological fitness and interaction stability are often more important than maximal metabolic output. Nevertheless, relatively few studies have evaluated EMS-derived mutants in plant-associated or soil-based systems, underscoring a persistent gap between laboratory-scale strain improvement and field-relevant agricultural outcomes.

Finally, while EMS-derived fungal mutants show promising potential for agricultural biotechnology, their deployment requires cautious evaluation beyond laboratory optimisation. As a random mutagenesis approach, EMS introduces numerous genome-wide mutations that may influence ecological fitness, stability, or unintended phenotypic traits if not rigorously validated (Hoyos-Manchado et al. 2020; Chhaya and Gupte 2019). Although EMS mutants are generated through classical mutagenesis rather than transgenic modification, responsible application in agricultural systems necessitates multi-generational stability testing, ecological fitness assessment, and consistent functional performance across diverse environmental conditions. Integrating greenhouse trials, soil microcosm assays, and field-relevant validation will therefore be essential to ensure that laboratory-optimised traits translate into robust and reliable performance in real-world agroecosystems (Meyer et al. 2016).

### Genetic studies

Across fungal systems, EMS mutagenesis has proven effective for generating the genetic variability needed to interrogate metabolic pathways and functionally relevant traits for sustainable agriculture (Jankowicz‐Cieslak and Till, 2016; Corbu et al. 2023). Multiple studies demonstrate that EMS exposure produces dense point-mutation profiles conducive to identifying regulatory control points underlying metabolite secretion, stress adaptation and plant-associated phenotypes (Mohd-Yusoff et al. 2015; Lakhssassi et al. 2020).

Experimental evidence across Aspergillus spp., Penicillium spp., and soil-derived fungi indicates that EMS-derived mutant libraries frequently yield enhanced extracellular enzyme activities, antibacterial metabolite production, and modified interaction phenotypes (Leonard et al. 2013; Shreya et al. 2019), as reflected in the studies summarized in Table 1. These applications have enabled researchers to explore fungal biochemical pathways without requiring prior genetic knowledge or transformation-dependent systems (Aklilu 2021).

However, most EMS-based genetic studies remain concentrated at the laboratory scale, often without long-term stability assessment or integration of genomic validation technologies (Chen et al. 2023).

Beyond its value for classical strain improvement, EMS mutagenesis presents important opportunities within agroecology, where fungi act as foundational ecological engineers. EMS-derived mutants with altered stress tolerance, secondary metabolite profiles, or plant-interaction traits could directly contribute to soil nutrient cycling, rhizosphere modulation, and enhanced plant resilience under climate stress. Beneficial fungi with enhanced saprophytic activity have the potential to improve organic matter turnover and soil structure, while altered secretion profiles could strengthen mutualistic interactions in mycorrhizal and endophytic systems. Likewise, generating EMS mutants with improved biocontrol capacity could reduce dependence on synthetic pesticides, aligning fungal biotechnology with low-input agricultural management. Critically, using EMS in this context shifts the focus from isolated laboratory traits toward holistic ecological function, where fungal performance is evaluated in environmental, community-level contexts. As such, EMS mutagenesis has the capacity not only to refine fungal metabolic output, but to actively shape agroecosystem resilience, sustainability, and productivity.

### Strain improvement

EMS mutagenesis has emerged as a strategic tool to generate fungal variants with enhanced metabolic performance and stress tolerance, and these traits can be leveraged to support sustainable agricultural systems (Salazar-Cerezo et al. 2023). Conventional strain improvement approaches often depend on targeted genetic modification or classical selection, which can be limiting when applied to non-model fungi or when incremental changes in metabolic output are required. EMS, by contrast, provides a means to generate broad allelic diversity that can then be screened for desirable traits.

Numerous EMS studies have reported improvements in cellulase, lipase and other hydrolytic enzyme activities, stress tolerance and pathogenicity attributes (Table 1), reflecting its utility as a rapid mutational strategy for strain optimisation (Ahmed et al. 2025; Adsul et al., 2007; Leonard et al. 2013; Zhu et al. 2016). While many applications have traditionally focused on industrial enzyme enhancement, emerging work highlights the potential of EMS to support ecologically beneficial traits, including host compatibility and biocontrol capacity. Integrating EMS mutagenesis with genomic analysis and staged phenotypic screening may therefore accelerate the development of resilient strains suitable for agricultural deployment.

### Pathogenicity studies

Across fungal pathogens, EMS mutagenesis has provided valuable insight into the genetic determinants of virulence, host specificity and stress adaptation (Wongwanich et al. 2017; Shreya et al. 2019). Studies across genera such as Beauveria, Aspergillus and Magnaporthe demonstrate that EMS-induced allelic diversity can alter pathogenicity-associated traits including thermotolerance, sporulation and infection efficiency, supporting its utility for dissecting complex host–fungus interactions (Knoll et al. 2011; Ahmed et al. 2025). These observations align with broader evidence that point mutations can reveal incremental or partial functional changes in pathogenic pathways that may be obscured using knockout-only approaches.

Across the literature, EMS-derived mutants often show either attenuation or enhancement of virulence phenotypes, suggesting that low-level mutational variation can shift infection outcomes in both directions depending on the genetic context (Wongwanich et al. 2017; Avramovska and Hickman, 2019). However, pathogenicity-focused EMS studies remain predominantly small-scale and isolate-specific, with limited replication, short-term infection assays and minimal genomic validation. Long-term trait stability, environmental performance, and ecological relevance are rarely assessed.

Advancing EMS-based pathogenicity research will therefore require integration with genome-wide mutation profiling, targeted allele mapping and environmentally relevant challenge assays, enabling clearer linkage between induced mutations, regulatory pathways and disease outcomes in agricultural contexts.

### Functional genomics

The genus, *Aspergillus*, consists of organisms that have agricultural, industrial, pathological, and pharmaceutical importance (Nayak et al. 2020). Several species are essential for healthy agricultural soils as they help solubilize phosphates, produce secondary metabolites that aid plant growth, and produce phytohormones (Hung and Rutgers 2016). *Aspergillus nidulans* has long been a useful model for identifying genes involved in the generation of secondary metabolites and universal regulators of the cell cycle and cytoskeleton (De Souza et al. 2013).

Through heterokaryon rescue De Souza et al. (2013) found the terminal phenotypes associated with 23 critical kinases after performing gene deletions of 128 *A. nidulans* kinases. The phenotypes associated with forty-three non-essential kinases were also found. Furthermore, they provide evidence for several kinase functions that were previously unknown, such as the morphogenesis Orb6 kinase signaling pathways, the septation initiation network, and cell wall integrity.

By creating a gene-dereplication strain of *A. nidulans*, another study found a novel secondary metabolite called aspercrytin (Chiang et al. 2016). The gene dereplication strain was developed through the deletion of gene clusters for the most highly expressed secondary metabolites’ pathways in *A. nidulans*. Through this work, they were also able to propose the potential biosynthetic pathway for this novel compound.

Since the 1920s, *Neurospora crassa* has been employed as a useful model organism in research pertaining to genetics, biochemistry, and development (Aramayo and Selker 2013). Due to its rapid growth, ease of propagation, and susceptibility to genome mutation, this organism continues to be a valuable model organism. Probst et al. (2014) were able to comprehensively characterize *Neurospora crassa’s* molybdenum cofactor (Moco) biosynthetic pathway through the deletion of all its biosynthetic genes. They annotated 5 genes for all enzymes involved in the pathway and compared it to the characterized *A. nidulans* pathways. Moco is an important cofactor as many enzyme activities, such as nitrate assimilation and the utilization of hypoxanthine as dependent on it.

Furfural is a compound that is found in high concentrations following the pretreatment of plant biomass for biofuel production and inhibits efficient fermentation by *Saccharomyces cerevisiae*. Feldman et al. (2019) searched for genetic attributes in *N. crassa* linked to furfural tolerance. They performed both hypothesis-based specific and unbiased mutations to this end and found mutants that were approximately 30% more tolerant to furfural exposure. The specific mutations showed that CRE-1 transcription factor, aldehyde dehydrogenase ahd-2 (NCU05580) and unbiased mutagenesis inactivation of two hypothetical proteins NCU02488 and NCU01407 increased tolerance to furfural.

### Comparative perspective: plant vs. fungal EMS frameworks

While EMS mutagenesis is deeply embedded in plant genetics, fungal studies remain comparatively fragmented. In plant systems, EMS efforts routinely involve large mutant libraries, structured screening pipelines, and integration with whole-exome or whole-genome sequencing platforms (Li et al. 2017; Lian et al. 2020; Chen et al. 2023). In contrast, fungal EMS research is typically represented by small, isolated case studies targeting limited numbers of mutants and single-trait screens, with few examples incorporating genome-wide mutation cataloguing or systematic validation (Gu et al. 2020; Ahmed et al. 2025). Whereas plant platforms benefit from standardised, population-scale workflows and curated datasets, fungal pipelines remain largely bespoke, low-throughput, and lack unified sequencing-based frameworks (Mohd-Yusoff et al. 2015; Fonseca et al. 2022). Consequently, the knowledge generated in fungi is uneven in scope and depth, despite comparable mutational potential.

These developments illustrate how EMS mutagenesis has expanded from strain-improvement studies into a versatile framework supporting functional genomics, fungal ecology, and agricultural innovation.

## Conclusion

Ethyl methanesulfonate has transitioned into a valuable and widely used mutagen in microbial research, underpinning advances across forward genetics, reverse genetics, and strain optimisation platforms. Its broad applicability, low cost, and high mutational density have facilitated the discovery of traits relevant to sustainable agriculture, including enhanced enzyme secretion, stress tolerance, and biocontrol potential.

However, the utility of EMS must be critically considered alongside its limitations. Challenges associated with mutation stability, phenotypic reversion, cytotoxicity at higher dosages, and mutational bias complicate downstream selection and may hinder the long-term performance of derived strains. Moreover, many EMS-based studies remain restricted to laboratory conditions, often lacking genomic validation, ecological assessment, or field-level performance testing.

Looking forward, integrating EMS mutagenesis with high-resolution sequencing technologies, systems-level phenotyping, and stability assessments will be essential to overcome these constraints. Such combined approaches will enhance causal mutation identification, improve mutant reliability, and enable more effective translation into agroecological applications. Thus, while EMS remains an important tool in fungal genetics, its continued refinement and critical integration with modern molecular platforms will determine its future contribution to sustainable agriculture.

## Data Availability

No datasets were generated or analysed during the current study.

## Code availability

Not applicable. No custom code or software was developed for this work.
